# Fourth-generation ceramic-on-ceramic THA results in improvements in midterm outcomes compared to third-generation THA but does not resolve noise problems: a cohort study of a single-hip system

**DOI:** 10.1186/s12891-019-2641-x

**Published:** 2019-05-30

**Authors:** Seung-Chan Kim, Young-Wook Lim, Woo-Lam Jo, Hyun-Woo Park, Sung-Bin Han, Soon-Yong Kwon, Yong-Sik Kim

**Affiliations:** 1 Department of Orthopaedic Surgery, Seoul St. Mary’s Hospital, College of Medicine, The Catholic University of Korea, Banpo-daero 222, Seocho-gu, Seoul 137-701 South Korea; 2 Department of Orthopaedic Surgery, Eunpyeong St. Mary’s Hospital, College of Medicine, The Catholic University of Korea, Seoul, South Korea

**Keywords:** Total hip arthroplasty, Ceramic-on-ceramic, Bearing, Outcome, Ceramic fracture, Noise

## Abstract

**Background:**

Using data from the Korean Hip Registry, we aimed to investigate mid-term clinical and radiographic outcomes, including the prevalence of periprosthetic joint infection (PJI), osteolysis, and component loosening or dislocation, and to analyze the incidence of bearing-related complications following modern ceramic-on-ceramic (COC) total hip arthroplasty (THA) using a single cementless hip system.

**Methods:**

Four hundred eighty-two patients (602 hips) who underwent Forte or Delta COC THAs with a single hip system and had a minimum 5-year follow-up were identified. The sample included 243 (50.4%) women and 239 (49.6%) men with a mean age of 50.6 years (range: 18–83 years). The Forte group comprised 310 hips, and the Delta group comprised 292 hips. The mean follow-up was 6.1 years (range: 5–10.2 years).

**Results:**

Cup orientation did not differ between groups. No hip had a PJI or osteolysis in either group. All acetabular components and all but two femoral components (in the Delta group) were well fixed. Dislocations occurred in six (1.9%) hips in the Forte group and one (0.3%) hip in the Delta group (*p* = 0.124). A total of nine (1.5%) revisions were performed. The 5-year survival rates for all-cause revisions were 98.4 and 98.6%, respectively. One (0.3%) ceramic head fracture occurred in the Forte group. Sixteen (5%) hips exhibited clicking and 6 (2%) hips had squeaking in the Forte group; 16 (6%) hips exhibited clicking and 5 (2%) hips had squeaking in the Delta group. Multiple regression analysis revealed that noise generation was unassociated with any factor.

**Conclusions:**

From the Korean Hip Registry data, THA with modern ceramic bearings showed encouraging results, with lower risks of PJI, osteolysis, and component loosening. In particular, Delta COC THA resulted in no PJI or ceramic fracture and had a reduced dislocation risk. However, associated noise remains a concern.

## Background

Ceramic bearing surfaces have been developed as an alternative to metal-on-polyethylene bearings in total hip arthroplasty (THA) in an attempt to reduce wear and improve implant longevity [1, 2]. More recently, ceramic-on-ceramic (COC) bearings have been suggested to be associated with reduced risks of dislocation [3, 4] and infection [5, 6]. Improvements in material properties, increased modular options, and concerns about trunnionosis have led to the increasing use of ceramic bearings in THA [7, 8]. In South Korea, in particular, the use of COC bearings has increased steadily over the past decade; the rate of use exceeded 80% in 2011 [9]. In addition, nearly 50% of all implanted ball heads registered with the American Joint Replacement Registry in 2014 were of the ceramic variety [7].

Despite the greater use of COC bearings over the last decade, major concerns persist regarding potential adverse events such as ceramic fracture [2, 6, 8, 10], noise generation [11–15], and a decreased positional range of error during liner insertion [10, 16]. These concerns, as well as the increased cost of the implant, remain the principal barriers to the wide adoption of ceramics, even after the introduction of a modern alumina matrix composite (AMC) ceramic (BIOLOX® Delta; CeramTec AG, Plochingen, Germany) to address some of the concerns raised with the pure alumina (PA) ceramic design (BIOLOX® Forte). Although the etiologies of ceramic fracture and noise generation are multifactorial, some previous studies have demonstrated that implant design, with regard to stem characteristics, geometry, and taper design or taper mismatch, can also have an impact on these problems unique to hard-on-hard bearings [7, 12]. However, to our knowledge, few studies have investigated the clinical and radiographic outcomes of the use of modern PA and AMC COC bearings in cementless THA with a single implant system with mid- to longer-term follow-up.

The purpose of this study was to report on (1) the mid-term clinical and radiographic outcomes; (2) the prevalence of periprosthetic joint infection (PJI), osteolysis, aseptic loosening of the component, and dislocation; and (3) the incidence of bearing-related complications, such as ceramic material fracture and noise generation, in a cohort of patients who underwent third- or fourth-generation COC THA using a single cementless hip system. In addition, we specifically sought to determine whether specific patient and/or surgical factors were associated with noise generation.

## Methods

The Korean Hip Registry (KHR) was launched by the Korean Hip Society in 2010 to improve outcome assessment and patient care after joint replacement surgery, and to promote research. All patients consented to the inclusion of their anonymized personal data in the KHR. Using the KHR, we identified 626 hips in 504 patients who received Forte or Delta COC bearings for primary THA with a single hip system at our institution between January 2007 and March 2012. All patient, surgical, and anesthetic information of interest and follow-up data obtained at each clinic visit were evaluated using our institution’s local joint database. The inclusion criteria consisted of a patient age ≥ 18 years and a minimum follow-up period of 5 years. We excluded patients with a diagnosis of femoral neck fracture; previous hip surgery; neurological disease; and significant involvement of the knee, ankle, or spine that limited walking. Of the 504 patients, 8 patients (9 hips) were lost to follow-up or had incomplete data and 3 patients (4 hips) died from causes unrelated to the procedure before the end of the 5-year follow-up period. In addition, 11 patients (11 hips) were excluded from the study (6 femoral neck fractures, 2 previous hip surgeries, 1 neurological disease, 1 bony ankylosis of the knee, and 1 severe spinal deformity), leaving 482 patients (602 hips) available for this retrospective analysis with a mean follow-up duration of 6.1 years (range: 5–10.2 years). This study was approved by the Institutional Review Board of Seoul St. Mary’s Hospital (approval No. KC17RESI0275) and all patients provided written informed consent.

The total sample comprised 243 (50.4%) women and 239 (49.6%) men with a mean age of 50.6 years (range: 18–83 years) at the time of surgery. The mean body mass index (BMI) was 23.4 (range: 16.8–39.3). Diagnoses were osteonecrosis of the femoral head in 322 (53%) hips, osteoarthritis secondary to hip dysplasia in 102 (17%) hips, primary osteoarthritis in 103 (17%) hips, post-traumatic arthritis in 14 (2%) hips, sequelae of pyogenic arthritis in 19 (3%) hips, sequelae of Legg-Calve-Perthes disease in 21 (4%) hips, rheumatoid arthritis in 17 (3%) hips, and ankylosing spondylitis in 4 (1%) hips.

The patients were divided into two groups based on the ceramic bearings implanted. The Forte COC group consisted of 248 patients (310 hips) who received 32-mm and 28-mm femoral heads, and the Delta COC group consisted of 234 patients (292 hips) who received 36-mm and 32-mm femoral heads. Patient demographics did not differ significantly between groups (Table 1).

**Table 1:** Patient demographics

Characteristics	Forte COC (*n* = 310)	Delta COC (*n* = 292)	*p* value
Number of patients (hips)	248 (310)	234 (292)	
Age^a^ (years)	50.7 (18–79)	50.5 (18–83)	0.838
Sex^c^			0.204
Male	116 (47)	123 (53)	
Female	132 (53)	111 (47)	
Affected side^b^			0.902
Right	163 (53)	155 (53)	
Left	147 (47)	137 (47)	
BMI^a^ (kg/m^2^)	23.3 (16.9–39.3)	23.5 (16.8–37.9)	0.461
Primary diagnosis^b^			0.895
ONFH	167 (54)	155 (53)	
Hip dysplasia	55 (18)	47 (16)	
Osteoarthritis	49 (16)	54 (18)	
Post-traumatic arthritis	9 (3)	5 (2)	
Sequelae of pyogenic arthritis	11 (3)	8 (3)	
Sequelae of LCP disease	9 (3)	12 (4)	
Rheumatoid arthritis	8 (2)	9 (3)	
Ankylosing spondylitis	2 (1)	2 (1)	
Cup size^a^ (mm)	53.9 (46–62)	54.4 (46–64)	0.324
Femoral head size^b^			
28 mm	87 (28)	–	
32 mm	223 (72)	43 (15)	
36 mm	–	249 (85)	
Duration of follow-up^a^ (years)	6.7 (5–10.2)	5.5 (5–7.4)	

^a^ The values are given as the average, with the range in parentheses

^b^ The values are given as the number of hips, with the percentage in parentheses

^c^ The values are given as the number of patients, with the percentage in parentheses

*COC* ceramic-on-ceramic, *BMI* body mass index, *ONFH* osteonecrosis of the femoral head, *LCP* Legg-Calve-Perthes

A single surgeon (the senior author) performed all operations using a posterolateral approach under general anesthesia. All hips in both groups received the same cementless hemispherical titanium cup (Bencox®; Corentec, Cheonan, Korea) with or without screws. The cup was plasma sprayed with microporous pure titanium with more than 30% porosity. One or two screws were used when the surgeon believed they would be helpful in acetabular component fixation, based on bone quality. According to the product specifications, a 32-mm-diameter BIOLOX® Forte ceramic liner (CeramTec AG) was inserted when the cup size was ≥52 mm, and a 28-mm BIOLOX® Forte ceramic liner was inserted when the cup size was < 52 mm, in the Forte COC group. A 36-mm or 32-mm-diameter BIOLOX® Delta ceramic liner was inserted in each hip in the Delta COC group based on the same criteria. A 32-mm BIOLOX® Forte ceramic head without a metallic sleeve was implanted in 223 (72%) hips and a 28-mm BIOLOX® Forte head was implanted in the remaining 87 (28%) hips in the Forte COC group. In the Delta COC group, a 36-mm BIOLOX® Delta ceramic head was implanted in 249 (85%) hips and a 32-mm BIOLOX® Delta head was implanted in the remaining 43 (15%) hips (Table 1). The femoral component was a cementless Bencox® stem (Corentec), a grit-blasted, micro-arc oxidized double-tapered wedge stem with a rectangular cross section and a fixed neck–shaft angle of 135°. Patients were instructed to begin walking on the first or second postoperative day with the assistance of a frame or two crutches, and were advised to use a walking aid for a period of 6 weeks.

Routine follow-up visits were scheduled at 6 weeks; 3, 6, and 12 months; and yearly thereafter. However, patients were allowed to return to the clinic regardless of the regular schedule if they encountered a problem or experienced complications with their hips. No patient was recalled specifically for the study, but some patients were contacted by telephone when scheduled follow-up visits were missed. Each clinic visit included radiographic evaluation and a physical examination, as well as the reporting of any relevant issues. Functional outcome assessment was performed with the use of the Harris Hip Score (HHS) preoperatively and at the time of the final follow-up. To obtain the incidence of noise, all patients were specifically asked if they had heard any noise from their hip at each follow-up visit or when contacted by phone. Patients who reported noise were asked to characterize it (clicking, squeaking, or other) and to try to reproduce it during the clinic visit. Each report of noise was recorded as an adverse event in the registry.

Radiographic evaluations were performed using standardized radiographs digitized into the picture archiving and communication system. A supine anteroposterior (AP) radiograph of the pelvis with both hips in 15° internal rotation and lateral views of each hip were obtained on the first postoperative day and at each subsequent visit. When taking plain radiographs, every effort was made to reduce anterior or posterior pelvic tilt throughout the study. The abduction and anteversion angles of the acetabular component, center of rotation (COR) in the horizontal and vertical planes [17], femoral/hip offset [17], and leg-length discrepancy (LLD) were measured as radiographic parameters after THA on 3-month standardized radiographs. Intraclass correlation coefficient values for intra-rater and inter-rater reliability were demonstrated to be excellent (all > 0.85) for these parameters in our previous studies [17, 18]. We used the method described by Lewinnek et al. [19, 20] for the measurement of anteversion. The postoperative LLD was assessed using the method of Woolson et al. [21]. The actual values for each measure were obtained via a calibration process using the size of the implanted femoral head. One independent observer who did not participate in the operations and was blinded to the patient’s information evaluated all radiographs.

The serial radiographs were analyzed to evaluate component loosening, osteolysis, PJI, periprosthetic fracture, and dislocation. Acetabular components were considered to be loose when a change in cup inclination > 4° or a change in cup migration > 4 mm was detected [22]. Femoral components were considered to be unstable when a progressive axial subsidence > 3 mm, a continuous radiolucent line > 2 mm, or a varus or valgus shift > 3° was observed [23]. Osteolytic lesions were assessed according to the criteria of Engh et al. [24]; lesions were recorded in the three zones described by DeLee and Charnley [25] on the acetabular side, and the seven zones described by Gruen et al. [26] on the femoral side. Stress shielding was graded on the radiographs at the last follow-up using Engh and Bobyn’s criteria [27]. Ceramic liner wear was calculated according to the method of Livermore et al. [28]. The total wear rate was determined by comparing the AP radiograph obtained 6 weeks after surgery with the one obtained at last follow-up. Measurements were made using a digital caliper with a resolution of 0.01 mm and calculations were subsequently adjusted with reference to the ceramic head diameter. The annual wear was calculated by dividing total wear by the years of follow-up. The position of the femoral stem was classified as neutral, varus (> 5° medial deviation), or valgus (> 5° lateral deviation) [29].

### Statistical analysis

Descriptive statistics included frequencies and percentages for categorical data, and means and standard deviations (SDs) for continuous data. Categorical data were compared between groups using the chi-squared test or Fisher’s exact test, and continuous data were compared using Student’s *t* test or the Mann–Whitney *U* test. A Kaplan–Meier survival analysis was performed with revision for any reason serving as one endpoint and revision due to aseptic loosening serving as the other endpoint. Multiple logistic regression analysis was used to examine possible associations of the following factors with noise generation: age, sex, BMI, bearing surface, cup abduction angle, anteversion angle, cup size, head size, and HHS at the final follow-up. Statistical analysis was performed using SPSS for Windows (ver. 21; SPSS Inc., Chicago, IL, USA), and the level of significance was set at *p* < 0.05.

## Results

Acetabular component orientation did not differ between groups (Table 2). The mean abduction angles were 41.8 ± 7.1° (range: 25–57°) in the Forte COC group and 42.1 ± 6.5° (range: 28–58°) in the Delta COC group (*p* = 0.522), and the mean anteversion angles were 15.8 ± 7.1° (range: 4–36°) and 16.2 ± 6.0° (range: 4–37°), respectively (*p* = 0.464). Comparison of the other radiographic parameters, such as femoral stem position, COR position, femoral offset, hip offset, and postoperative LLD, also revealed no significant difference between groups (Table 2).

**Table 2:** Radiographic results following ceramic-on-ceramic THA

Parameters	Forte COC (n = 310)	Delta COC (n = 292)	*p* value
Acetabular component orientation^a^ (°)			
Abduction angle	41.8 (25–57)	42.1 (28–58)	0.522
Anteversion angle	15.8 (4–36)	16.2 (4–37)	0.464
Femoral component position^b^			0.669
Neutral	302 (97)	286 (98)	
Varus	8 (3)	6 (2)	
Valgus	0 (0)	0 (0)	
COR position^a^ (mm)			
Vertical	16.9 (9.4–23.7)	16.8 (9.2–24.0)	0.733
Horizontal	27.7 (20.6–35.3)	27.5 (20.1–35.2)	0.681
Femoral offset^a^ (mm)	39.3 (32.8–49.5)	39.7 (33.0–49.8)	0.541
Hip offset^a^ (mm)	67.0 (53.5–79.6)	67.2 (54.9–79.9)	0.839
Leg-length discrepancy^a^ (mm)*	2.6 (0–9.8)	2.5 (0.1–11.2)	0.677

^a^ The values are given as the average, with the range in parentheses

^b^ The values are given as the number of hips, with the percentage in parentheses

* Absolute values of leg-length discrepancy were obtained

*THA* total hip arthroplasty, *COC* ceramic-on-ceramic, *COR* center of rotation

During a minimum follow-up period of 5 years, no hip in either group showed osteolysis, PJI, or measurable wear. All acetabular components in both groups showed osseointegration with radiographic evidence of bone ingrown stability. All hips in the Forte COC group and all but two hips in the Delta COC group showed osseointegration of the femoral components. These two (0.7%) hips exhibited aseptic loosening of the stem due to the insertion of undersized stems. Dislocation occurred in six (1.9%) hips (four with 28-mm heads, two with 32-mm heads) in the Forte COC group and one (0.3%) hip (with a 36-mm head) in the Delta COC group (*p* = 0.124). Among those, six hips were treated successfully with closed reduction and the subsequent application of an abduction brace, and the remaining hip required acetabular cup revision for recurrent dislocation. These patients have not experienced any further dislocation. A total of nine (1.5%) revisions were performed over the course of the study (five [1.6%] in the Forte COC group and four [1.4%] in the Delta COC group): five revisions (three in the Forte COC group and two in the Delta COC group) were performed due to Vancouver type B2 periprosthetic femoral fracture, two revisions (in the Delta COC group) were performed due to stem loosening, one revision (in the Forte COC group) was performed due to ceramic head fracture, and one revision (in the Forte COC group) was performed due to recurrent dislocation.

**Fig. 1:**
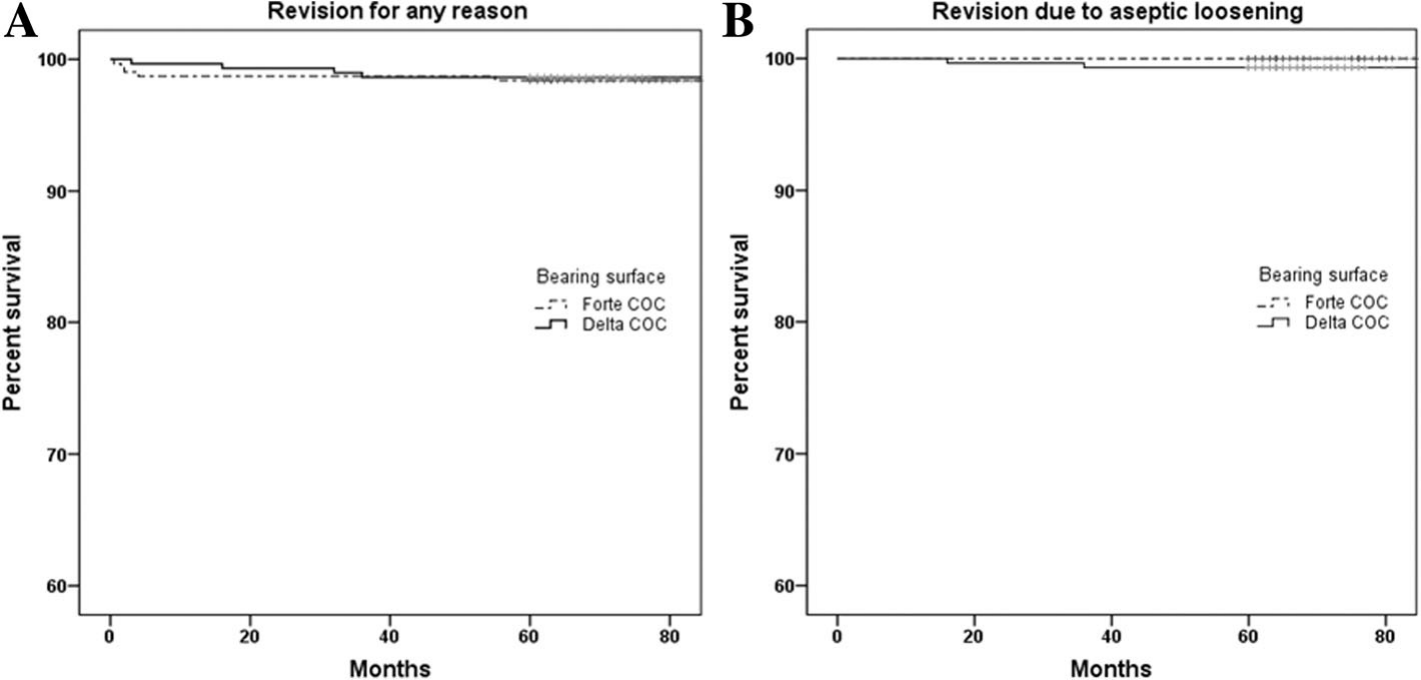
Kaplan–Meier survival curves for the Forte ceramic-on-ceramic (COC) and Delta COC groups with revision for any reason (**a**) and revision due to aseptic loosening (**b**) serving as the endpoints

Kaplan–Meier survival rates for all-cause revision at a minimum of 5 years were 98.4% (95% confidence interval [CI], 97.0–99.8) in the Forte COC group and 98.6% (95% CI, 97.2–99.9) in the Delta COC group (Fig. 1a). The survival rates for revision due to aseptic loosening were 100% in the Forte COC group and 99.3% (95% CI, 98.3–100) in the Delta COC group (Fig. 1b).

**Fig. 2:**
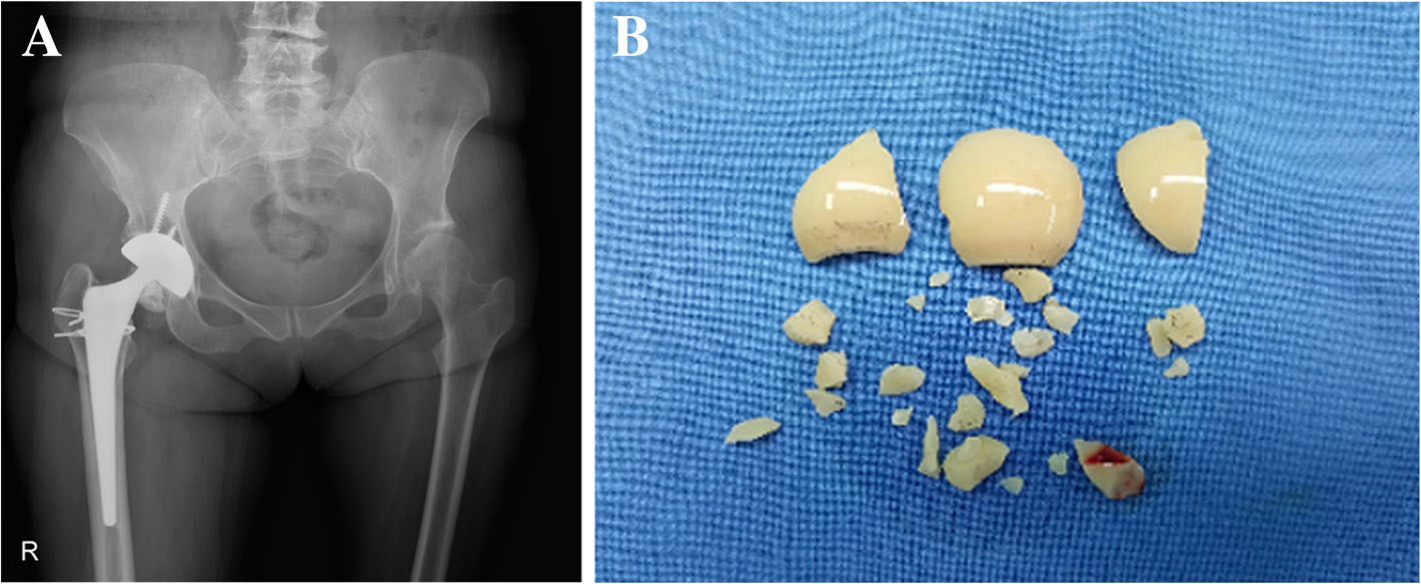
**a** An anteroposterior radiograph of the right hip of a 57-year-old woman showing a fracture of the alumina Forte femoral head (28 mm, short neck) at 4.5 years after total hip arthroplasty. The patient noted a repeated squeaking sound during walking that began at 21 months after the index surgery, and subsequently fell on the stairs 2 days before the fracture developed. **b** The retrieved fractured head, showing many variously sized fragments

One (0.3%) ceramic fracture occurred in an alumina Forte 28-mm short-neck femoral head; the patient was a 57-year-old woman with a history of trauma and repeated squeaking before the fracture occurred, and she underwent revision surgery at 4.5 years after the index THA (Fig. 2). The acetabular cup was fixed and positioned well (inclination, 40.2°; anteversion, 14.5°). A new Delta COC bearing including a ceramic head with a titanium sleeve (BIOLOX® option) was inserted after complete synovectomy and thorough debridement. No (0%) ceramic head or liner fracture occurred in the Delta COC cohort, and no liner dissociation was observed in either group.

In the entire cohort, 43 (7%) patients reported some type of noise in the hip. In the Forte COC group, 16 of 310 (5%) hips exhibited clicking and 6 (2%) hips exhibited squeaking. In the Delta COC group, 16 of 292 (6%) hips exhibited clicking and 5 (2%) hips exhibited squeaking. These noises were not reproducible and were not associated with pain or functional impairment except the patient who developed the subsequent Forte head fracture. Clicking was first reported at 2.8 ± 1.2 years (range: 0.5–5.8 years) after surgery in the Forte COC group and at 2.4 ± 1.3 years (range: 0.2–5 years) in the Delta COC group, and squeaking started on average at 2.2 ± 1.1 years (range: 0.9–4.4 years) after surgery in the Forte COC group and at 2.1 ± 1.4 years (range: 0.4–.6 years) in the Delta COC group. No difference in cup orientation was found between patients with and without noise: the mean abduction angles were 41.0 ± 6.5° (range: 28–53°) in patients experiencing noise and 42.0 ± 6.9° (range: 25–58°) in those without noise (*p* = 0.338), and the mean anteversion angles were 16.4 ± 7.1° (range: 5–30°) and 16.0 ± 6.6° (range: 4–37°), respectively (*p* = 0.674). The mean HHS for patients experiencing noise was 93.7 ± 6.3 points (range: 75–99 points), and no hip was revised or is currently awaiting revision for a noise problem. Multiple logistic regression analysis revealed that noise generation was not associated with age (*p* = 0.599), sex (*p* = 0.615), BMI (*p* = 0.627), bearing surface (*p* = 0.727), cup abduction angle (*p* = 0.321), anteversion angle (*p* = 0.954), cup size (*p* = 0.440), head size (*p* = 0.749), or HHS at the final follow-up (*p* = 0.523). Moreover, no association was found between these factors and the incidences of clicking and squeaking, respectively.

Mean preoperative and postoperative HHSs did not differ between groups. The mean preoperative HHSs were 42.8 ± 11.8 points (range: 10–57 points) in the Forte COC group and 42.3 ± 12.7 points (range: 15–58 points) in the Delta COC group (*p* = 0.556). These scores improved to 93.9 ± 6.4 points (range: 67–100 points) and 94.1 ± 5.7 points (range: 70–100 points), respectively (*p* = 0.682), at the final follow-up.

## Discussion

Long-term issues with THA are linked to bearing surface wear and particle-induced osteolysis [30]. Strategies aimed at reducing wear and osteolysis have been focused on improving the material properties, as well as developing an alternative, e.g., ceramic, bearing surface [31]. Despite the increasing use of COC bearings in THA over the past decade, concerns regarding this bearing surface, including ceramic head or liner fracture, noise generation, and malseating of the liner, remain. However, few reports have described the clinical and radiographic outcomes following modern PA and AMC COC THAs using a single cementless hip system in a large cohort of patients with mid- to longer-term follow-up. Therefore, we investigated the mid-term clinical and radiographic outcomes, including the prevalence of PJI, osteolysis, component loosening, and dislocation; analyzed the incidence of bearing-related complications, such as ceramic fracture and noise generation; and determined the existence of specific patient or surgical factors associated with noise generation using data from the KHR.

Many previous studies regarding third- and fourth-generation ceramic bearings have found little or no osteolysis [1, 4, 10, 11, 32, 33]. In the present study, we also found no evidence of acetabular or femoral osteolysis at the final follow-up. The survival results were encouraging and similar to those of previously published reports on modern COC bearings [1, 8, 10, 31–35]. For Forte COC bearings with alumina 28-mm heads, Kim et al. [33] reported a 99.7% 20-year overall survival rate for all-cause revision. In addition, D’Antonio et al. [31], in their multi-surgeon, multi-center study, reported 10-year survival rates of 97.9% for System I and 95.2% for System II; Lee et al. [34] reported a 10-year survival rate of 99%, and Murphy et al. [35] reported a 9-year survival rate of 96%. For the Delta COC bearings, Hamilton et al. [1] reported 5-year survival rates of 97.7% for the 28-mm COC arm and 97.3% for the 36-mm COC arm, and Kim et al. [32] reported a 13-year survival rate of 99.7%, predominantly for the 36-mm femoral head. Survival rates in our series with regard to all-cause revision were 98.4% for the Forte COC cohort and 98.6% for the Delta COC cohort at 5 years. PJI is a disastrous and challenging complication occurring after THA [5, 36]. In the present study, no hip had an early or late PJI in either the Forte or Delta COC group. Some recent reports have suggested that infection is associated with bearing surface type [5, 6, 37, 38]. Pitto et al. [5], in an analysis of New Zealand Joint Registry data over a 15-year period, suggested that COC bearings are associated with a lower risk of PJI compared with ceramic-on-polyethylene, metal-on-polyethylene, and metal-on-metal bearings, but emphasized that these findings must be considered to be extremely preliminary. The authors found no difference in the rate of early PJI in the first 6 months among bearing surfaces whereas COC bearings were associated with a lower risk of revision for PJI over the entire period of observation. Our results also support these preliminary observations. Although the whole issue of PJI is complex and multifactorial, these results are likely to be related to the material properties of the ceramics. Ceramic bearings produce lower wear and their debris is more inert than that arising from any other bearing surface [35, 39–42]. Thus, a smaller amount of periprosthetic debris and subsequently reduced local tissue reaction can result in a protective effect against infection after THA [43].

The risk of dislocation after THA is also related to the bearing couples, although the cause of dislocation is too multifactorial [3, 4]. Hernigou et al. [3] found that, with no difference in cup orientation, COC bearings decreased the risk of dislocation, especially late dislocation, compared with ceramic-on-polyethylene bearings. They suggested that the reasons for the lower rate of dislocation with COC bearings were attributable to the more fibrous, thick capsule and reduced fatty atrophy in the periarticular muscles observed at the time of the revision, and that these differences might occur as a result of different biological responses to wear byproducts generated by different bearing surfaces [3, 4]. Other authors similarly reported lower dislocation rates in THA using Delta ceramics, ranging from 0.5 to 1.1% [10, 44, 45]. We found a lower dislocation rate (0.3%) in the Delta COC cohort, treated predominantly with 36-mm heads, than in the Forte COC cohort (1.9%), although this difference was not significant (*p* = 0.124). This finding is consistent with results reported in the literature (3.4% for the 28-mm COC vs. 1.8% for the 36-mm COC) [1]. The use of a larger femoral head with Delta ceramics combined with the lower wear rate would provide a major advantage, including improved stability, reduced impingement, and, consequently, a lower dislocation rate [8].

Despite advances in the manufacture of modern ceramics, ceramic material fracture remains a major concern. Recent results reported in the literature have demonstrated that the Delta ceramic is more fracture resistant than the Forte ceramic and, thus, that it has significantly reduced the risk of fracture [1, 7, 46, 47]. Massin et al. [46] performed a systematic review of the literature and reported head fracture rates of 0–10% with the BIOLOX® Forte ceramic, with a median close to zero. According to the French ceramic experience, the rates of head fracture were 0.18% with the Forte ceramic and 0.0013% with the Delta ceramic, which represent a 100-fold difference in favor of the Delta ceramic, whereas the manufacturer’s data (CeramTec) revealed a 20-fold difference (0.021% with Forte vs. 0.001% with Delta) [7, 46]. In contrast, the fracture rate of liners has remained stable at approximately 0.03 to 0.08%. We observed one (0.3%) fracture of a 28-mm short-neck alumina Forte head at 4.5 years after the index THA, and no ceramic fracture in the Delta COC cohort. Our findings are consistent with those of Kim et al. [32], who identified no Delta ceramic head or liner fracture at a mean of 13.1 years of follow-up. Ceramic fractures are usually associated with specific events, such as trauma, hip dislocation, or cup malposition (i.e., excessively abducted or anteverted cup angle) [7]. The patient in the current study experienced painful squeaking during her usual activities from 21 months after the index surgery and subsequently had a traumatic event, although the cup orientation was within the normal range. Abdel et al. [48] also found that painful squeaking following COC THA in four patients was related to ceramic liner fractures. They emphasized the need for a more thorough investigation, as squeaking was associated with increased pain.

Noise, especially squeaking, after THA has become a particular issue with ceramic bearings [11, 49]. Overall, the prevalence of squeaking has been reported in the English literature to range from 2 to 21% [11, 13, 48]; Stanat et al. [12] determined an average incidence of 2.4% in their meta-analysis. In the current study, the overall incidence of noise of any type was 7%, with the most common type of noise being clicking. Squeaking was reported in 2% of hips in each group, with no hip being revised due to this phenomenon. Although our results are similar to those of previous reports [11, 32] on modern ceramic bearings, the 2% incidence of squeaking in our study was lower than the rate of 7.5% published recently by Hamilton et al. [1]. The reason for this difference is unclear, but may be due to the influence of lower BMI in our series compared with Westerners. Although the exact etiology of squeaking remains uncertain, cup malposition, edge loading of components, decreased lubrication between the bearing surfaces, and metal transfer to the femoral head have been implicated as contributing factors [11]. In a meta-analysis, Stanat et al. [12] found BMI to be the only associated patient factor. Walter et al. [49] reported that cup malposition was associated with squeaking. In contrast, other authors [11, 13] did not find this association. These results are consistent with our findings, and we were unable to identify any factor that significantly affected noise generation.

This study has several limitations. First, despite the relatively large cohort compared with previously published studies [8, 10, 31, 34, 35, 45], our sample was not large enough for subgroup analyses of factors such as ceramic fracture or noise generation. In addition, the utilization of ceramic head size could not be matched between groups and the two cohorts were not comparable in the ceramic head size. In accordance with the manufacturer’s guidelines, 28 mm and 32 mm heads were only available for Forte ceramic bearings, and 32 mm and 36 mm heads for Delta ceramic bearings. The utilization of smaller heads in the Forte COC cohort can be a cause of concern regarding ceramic fracture (0.3% vs. 0%, *p* = 1.000) and dislocation (1.9% vs. 0.3%, *p* = 0.124) although not significantly different in this study. Second, the study was conducted using a single institution’s series from the registry database because data utilization for multicenter studies is not yet available in the KHR. However, we believe that the homogeneity of the current study, including the use of the same implant, surgeon, and surgical approach, can mitigate this shortcoming. Moreover, during the study period, we used only COC bearings for all primary THAs, regardless of patient age; therefore, selection bias may have been avoided. Third, although cup abduction and anteversion angle were measured, stem anteversion was not assessed. Moreover, computed tomography was not performed to evaluate osteolysis. However, our findings from standardized plain radiographs, which were assessed rigorously according to the criteria of Engh et al. [24], were similar to other published results. Fourth, we did not evaluate patient-reported outcome measures or activity levels or the influence that audible sounds had on them. Finally, this study included only East Asians with a mean BMI of 23.4 kg/m^2^. Therefore, these findings may not be generalizable to a Western population or to highly obese patients.

## Conclusion

According to the KHR data, cementless primary THA with modern ceramic bearings showed encouraging results with lower risks of PJI, osteolysis, and component loosening. In particular, Delta COC THA resulted in no PJI or ceramic fracture and had a reduced risk of dislocation. However, noise generation remains a concern.

## Data Availability

The datasets used and analyzed during the current study are available from the corresponding author on reasonable request.

## Abbreviations

AMC: Alumina matrix composite
BMI: Body mass index
COC: Ceramic-on-ceramic
COR: Center of rotation
HHS: Harris hip score
KHR: Korean Hip Registry
LLD: Leg length discrepancy
PA: Pure alumina
PJI: Periprosthetic joint infection
SD: Standard deviation
THA: Total hip arthroplasty
